# Impact of mask use on facial emotion recognition in individuals with subclinical social anxiety: an eye-tracking study

**DOI:** 10.1186/s41235-025-00635-4

**Published:** 2025-06-02

**Authors:** Jackie Wai Yi Wo, Weiyan Liao, Janet Hui-wen Hsiao

**Affiliations:** 1 Department of Psychology, The University of Hong Kong, Hong Kong SAR, China; 2 Division of Social Science, The Hong Kong University of Science and Technology, Hong Kong SAR, China; 3 Department of Computer Science & Engineering, The Hong Kong University of Science and Technology, Hong Kong SAR, China

**Keywords:** Social anxiety, Emotion recognition, Mask, Eye movement, EMHMM

## Abstract

Previous studies suggested that social anxiety is associated with interpretation bias, theory of mind deficit, and eye gaze avoidance when identifying facial emotions. We tested the hypothesis that socially anxious individuals would be more affected by mask use during facial emotion recognition. 88 healthy undergraduates with various levels of social anxiety were invited. Participants judged the emotions of masked and unmasked facial expressions. Eye Movement Analysis with Hidden Markov Models was used to analyze participants’ eye movement patterns during the task. Potential confounders including gender, depressive symptoms, stress, and executive planning ability were controlled for in the analyses. Results failed to support our hypothesis. Instead, higher social anxiety was associated with higher accuracy rates for angry and fearful faces and lower false alarm rates for sad faces. Eye movement patterns were similar across social anxiety levels. Interestingly, an exploratory moderation analysis revealed that an increase in using a more eye-centered strategy due to mask use was significantly associated with a larger drop in accuracy rate for fearful faces among individuals with higher social anxiety, while non-significantly associated with a smaller drop among individuals with lower social anxiety. Thus, our study indicates social anxiety, at least at subclinical levels, may be associated with a generally heightened sensitivity to negative emotions. However, such heightened sensitivity diminishes if they switch to a more eye-centered strategy when viewing masked facial emotions. Potential mechanisms and implications were discussed.

**Supplementary Information:**

The online version contains supplementary material available at 10.1186/s41235-025-00635-4.

## Significance statement

Social anxiety levels in the general population surged during the COVID-19 pandemic. At the same time, facial emotion recognition could be affected by mask use. It is thus important to understand whether the use of face masks may pose additional challenges to socially anxious individuals in facial emotion recognition. Our results paint a nuanced picture of cognitive processes involved in facial emotion recognition in individuals with subclinical social anxiety: individuals with high levels of social anxiety did not suffer a greater impairment in emotion recognition on masked faces compared with those with low levels of social anxiety. Instead, they had a generally heightened sensitivity to negative emotions regardless of mask use. However, their sensitivity would diminish if they switched their attention more to the eyes on masked faces. Therefore, covering lower faces with masks per se does not impair emotion recognition of socially anxious individuals to a greater extent, but rather the increase in eye contact in response to masks does. The increased salience of eye gaze due to masks may heighten distress in socially anxious individuals during eye contact, leading to the disruption of downstream emotion processing.

## Introduction

Social anxiety (SA) is characterized by the fear of being evaluated by others in social situations (Leary & Kowalski, 1995). The level of SA is considered to be a continuum, with social anxiety disorder (SAD) being at the most severe end. Patients with SAD suffer from intense distress in social situations and exhibit maladaptive avoidance behaviors, leading to significant functional impairment in daily life (American Psychiatric Association, 2022). As one of the most common anxiety disorders, SAD has a lifetime prevalence estimate of 11–12% in the US (Kessler et al., 2012; Ruscio et al., 2008). Moreover, up to 20% of the population who do not meet the diagnostic threshold of SAD experience symptoms of subclinical (i.e. subthreshold) social anxiety (Merikangas et al., 2002; Stein et al., 1994). Despite its greater prevalence than SAD (Johnson et al., 2022) and its considerable burden on functioning (Fehm et al., 2008), whether the mechanisms of subclinical social anxiety are distinct from SAD and its impact on the population remain less understood.

### Sociocognitive biases of social anxiety

Cognitive models have suggested the symptoms of social anxiety are attributed to impaired processing of social stimuli, especially facial expressions (e.g. Clark & Wells, 1995; Heimberg et al., 2014; for a recent review, see Rozen & Aderka, 2023). Facial expressions are important social signals that convey positive (e.g. acceptance) and negative evaluations (e.g. rejection) of others (Ekman & Friesen, 1969). Current literature has proposed several sociocognitive biases in people with high levels of SA that potentially impair facial expression processing, including (1) interpretation bias, (2) theory of mind deficit, and (3) eye gaze avoidance.

First, converging evidence has suggested that people with high levels of social anxiety are likely to display interpretation bias by showing more false alarms of negative emotions on ambiguous facial expressions. Recent studies have directly examined interpretation bias on faces by artificially manipulating facial expression ambiguity using morphing techniques. The first line of morphing studies manipulated the ambiguity of facial emotions by showing participants a face slowly morphing from a neutral expression (0% intensity) to a full-blown emotional expression (100% intensity). SAD patients were found to misinterpret neutral emotions as anger more often than healthy controls (Bell et al., 2011). Likewise, subclinically high SA individuals were found to misinterpret low-intensity disgust expressions as contempt (Heuer et al., 2010). These findings suggest social anxiety is associated with threat-related interpretation bias when viewing low-intensity facial expressions. The second line of research manipulated the ambiguity of emotions by blending facial expressions of opposite emotions. Using this method, Maoz et al. (2016) and Prieto-Fidalgo et al. (2023) found both subclinically and clinically SA individuals tended to judge angry-happy blends as anger.

Other research suggests that social anxiety may be related to an impaired theory of mind, although mixed evidence has been found. Theory of mind (ToM) refers to the ability to accurately understand one’s mental states, including emotions, and how mental states can influence one’s behaviors (Baron-Cohen et al., 1985). The majority of studies using the Reading the Mind in the Eyes Test (RMET; Baron-Cohen et al., 2001), a test in which participants have to identify the displayed mental state from only a pair of eyes, found that SAD patients made more errors in RMET than healthy or non-SAD counterparts (Hezel & McNally, 2014; Maleki et al., 2020; Öztürk et al., 2022; Washburn et al., 2016). These findings suggest the impairment in decoding others’ mental states from faces and the subsequent intensified uncertainty about social situations may contribute to the development of social anxiety (O’Toole et al., 2013). However, studies using subclinical samples have shown mixed results. Although a well-powered study by Alvi et al. (2020) found RMET impairment in subclinically SA individuals, two other studies found that subclinically SA individuals exhibited higher accuracy in affective ToM tasks. For example, Sutterby and colleagues (2012) found that subclinically high SA females performed better, instead of worse, in RMET than low SA females. In another ToM task, subclinically high SA individuals were also more accurate in inferring affective mental states based on one’s gaze direction and facial expression (Tibi-Elhanany & Shamay-Tsoory, 2011). The inconsistent results in the subclinical population may suggest a different ToM mechanism in subclinical levels of social anxiety, as compared with the clinical population.

Finally, attentional avoidance of direct eye gaze may also impair SA individuals’ facial emotion recognition. Eyes are considered one of the most important diagnostic features for emotion recognition (e.g. Schurgin et al., 2014; Wegrzyn et al., 2017). However, direct eye gaze is considered a threatening stimulus in the SA population, which is suggested by their heightened physiological responses in terms of heart rate acceleration (Wieser et al., 2009) and skin conductance responses (Myllyneva et al., 2015). SAD patients also displayed greater neural activation in the fear circuitry including the amygdala, insula, and anterior cingulate cortex in response to direct eye gaze (Schneier et al., 2009). Eye gaze avoidance, therefore, can act as a strategic safety behavior attempting to alleviate the elicited distress (Clark & Wells, 1995). Using the single-face free-viewing paradigm, researchers have consistently found that SAD patients showed fewer and shorter fixations to the eyes on positive and negative emotional faces (Horley et al., 2003, 2004; Moukheiber et al., 2010, 2012; Staugaard & Rosenberg, 2011; Weeks et al., 2013, 2019; c.f. Dalmaso et al., 2022 for eye contact avoidance in other anxious populations), generally with moderate effect sizes (for a review, see Günther et al., 2021).

### Effect of surgical masks on facial emotion recognition with social anxiety

The COVID pandemic has aggravated social anxiety in the general population, as longitudinal studies showed significant increases in social anxiety levels among adolescents and adults in the community (Hawes et al., 2022; Juvonen et al., 2022). At the same time, mask mandates may have introduced additional challenges for social interaction in the general population with a rising level of social anxiety, even though most may not reach the clinical threshold.

Indeed, the burden of surgical masks to facial emotion processing may interact with the sociocognitive biases associated with social anxiety, potentially leading to greater impairments in high SA individuals. First, as surgical masks restrict visible facial features solely to the eye region, substantial studies with diverse cultural and age samples have shown that people became less accurate when identifying emotions on masked faces, even though the extent of impairment on specific emotions was left inconclusive (e.g. Carbon, 2020; Carbon et al., 2022; Kim et al., 2022; Marini et al., 2021; Parada-Fernández et al., 2022). However, as evidenced in RMET studies, ToM deficits may further impair high SA individuals’ abilities to infer emotions solely from the eye region (Alvi et al., 2020; Hezel & McNally, 2014; Maleki et al., 2020; O’Toole et al., 2013; Öztürk et al., 2022; Washburn et al., 2016), resulting in even lower accuracy rates when recognizing masked facial emotions as compared to low SA individuals. Second, surgical masks significantly increase facial expression ambiguity, as consistent findings have shown that people perceived lower intensity of the intended emotion and higher intensity of the unintended emotions from masked faces (Pazhoohi et al., 2021; Tsantani et al., 2022). Given this, interpretation bias may prompt high SA individuals to interpret the ambiguous expressions on masked faces in a negative light, resulting in even higher false alarms of negative masked emotions compared with low SA counterparts.

Hence, in an exploratory review, Saint and Moscovitch (2021) have suggested cognitive bias modifications to mitigate the potential impact of masks on socially anxious individuals. However, evidence showing high SA individuals having greater impairments in recognizing emotions on masked faces has been limited, while some research has been done in populations with alexithymia (Fuchs et al., 2024) and autism (Pazhoohi et al., 2021).

### Eye movement analysis with hidden markov models

Previous studies have suggested high SA individuals present eye gaze avoidance behavior. However, little is known about whether eye gaze avoidance would lead to a larger impairment of facial emotion recognition on masked faces. To examine this, the current study adopted the eye movement analysis with hidden Markov models approach (EMHMM; Chuk et al., 2014, 2017). The EMHMM is a data-driven, machine-learning-based approach that provides a quantitative measure of eye movement patterns, which has been previously used in facial emotion recognition (Zhang et al., 2019; Zheng & Hsiao, 2023), face recognition (e.g. An & Hsiao, 2021; Chuk et al., 2014; Hsiao et al., 2022), and single-face free-viewing tasks (Chan et al., 2020a, 2020b). Using this approach, an individual’s eye movements were summarized using a hidden Markov model (HMM), including both person-specific regions of interest (ROIs) and transition probabilities among the ROIs. Subsequently, representative eye movement patterns among participants can be discovered through clustering individual HMMs, and dissimilarity between individual HMMs can be quantified by the log-likelihood of the individual’s data given the representative patterns. Previous studies have consistently discovered two representative face-viewing strategies: an “eye-centered” strategy that focuses more on the eye region, and a “nose-centered” strategy that focuses on the center of the faces more (Chan et al., 2020a, 2020b; Zhang et al., 2019; Zheng & Hsiao, 2023). In the context of social anxiety, previous research has interpreted the extent to which high SA individuals adopt the nose-centered strategy relative to the eye-centered strategy as their tendency to avoid direct eye gaze (Chan et al., 2020a). In other words, high SA individuals may be less likely to adopt an eye-centered strategy if they present eye gaze avoidance behavior.

In a recent study, Hsiao and colleagues (2022) used EMHMM to examine the differences in eye movement patterns of healthy participants when recognizing masked and unmasked faces, and how the eye movement pattern differences were associated with performance differences. The authors found that a smaller increase in using the eye-centered strategy was associated with a larger impairment when recognizing masked faces compared with unmasked faces. Accordingly, we hypothesized that a smaller increase in using the eye-centered strategy, i.e. more eye gaze avoidance, would also be linked to a larger impairment in facial emotion recognition of masked faces.

### Current study

As social anxiety levels in the general population have surged since the beginning of the COVID-19 pandemic (Hawes et al., 2022; Juvonen et al., 2022), this study aimed to investigate emotion recognition performance of individuals with subclinical social anxiety when viewing masked faces and examine their eye movement patterns using hidden Markov models. Using an undergraduate sample, this study aimed to understand how masks interact with the sociocognitive mechanism in subclinical social anxiety and whether cognitive bias modifications suggested by researchers (Saint & Moscovitch, 2021) should be applied not only to SAD patients, but the larger subclinical population.

Since participants’ levels of depression (Krause et al., 2021), stress (Daudelin-Peltier et al., 2017) as well as executive function abilities (e.g. Circelli et al., 2013; Zheng & Hsiao, 2023) may influence their recognition performance and eye movement behaviors, these were measured as control variables while we made the following hypotheses:

a) Given the consistent evidence of interpretation bias in both clinical (Bell et al., 2011; Maoz et al., 2016) and subclinical SA (Heuer et al., 2010; Prieto-Fidalgo et al., 2023), we hypothesized that a higher level of subclinical SA would be associated with a larger increase in false alarm rate of negative emotions in masked conditions, where facial expressions became more ambiguous.

b) Given the predominant evidence of theory of mind deficit in inferring mental states from the eyes in clinical (Hezel & McNally, 2014; Maleki et al., 2020; Öztürk et al., 2022; Washburn et al., 2016) and subclinical SA (Alvi et al., 2020), we hypothesized that a higher level of subclinical SA would be associated with a larger drop in accuracy rate and a larger increase in reaction time in masked conditions, where reliance on the eye region was necessary for recognizing emotions.

c) Given the consistent evidence of SAD patients showing eye contact avoidance (Horley et al., 2003, 2004; Moukheiber et al., 2010, 2012; Staugaard & Rosenberg, 2011; Weeks et al., 2013, 2019), we hypothesized that a higher level of subclinical SA would be associated with a greater tendency to adopt a more nose-centered viewing strategy in both masked and unmasked conditions.

d) Given the previous finding (Hsiao et al., 2022), we hypothesized that a smaller increase in using the eye-centered viewing strategy from unmasked to masked conditions would be associated with greater impairment in emotion recognition performance.

## Method

### Participants

Eighty-eight Chinese participants (Mean age ± SD: 21.2 ± 1.2, 54 females: 61%) were recruited based on the following criteria: (i) aged 18 or above, (ii) undergraduate students at Hong Kong universities, (iii) with normal or correct-to-normal vision, (iv) capable of reading traditional Chinese, and (v) with no self-reported current or past mental illnesses. This study was approved by the Human Research Ethics Committee, The University of Hong Kong (HREC number: EA200090).

Power analyses were performed. According to a simulation using the R packages lme4 and Smir, with the mean and effect size estimates from a previous study (Carbon, 2020), the required number of participants for a mixed-effects model with SA level, mask use, and emotion as fixed-effect predictors and participant as random intercept (power = 80%; *α* = 0.05) was 41. According to the calculation using G*Power 3.1, a minimum of 84 participants was needed to detect medium two-tailed correlations (*r* = 0.3, power = 80%; *α* = 0.05).

### Materials

#### Facial emotion recognition task

**Fig. 1:**
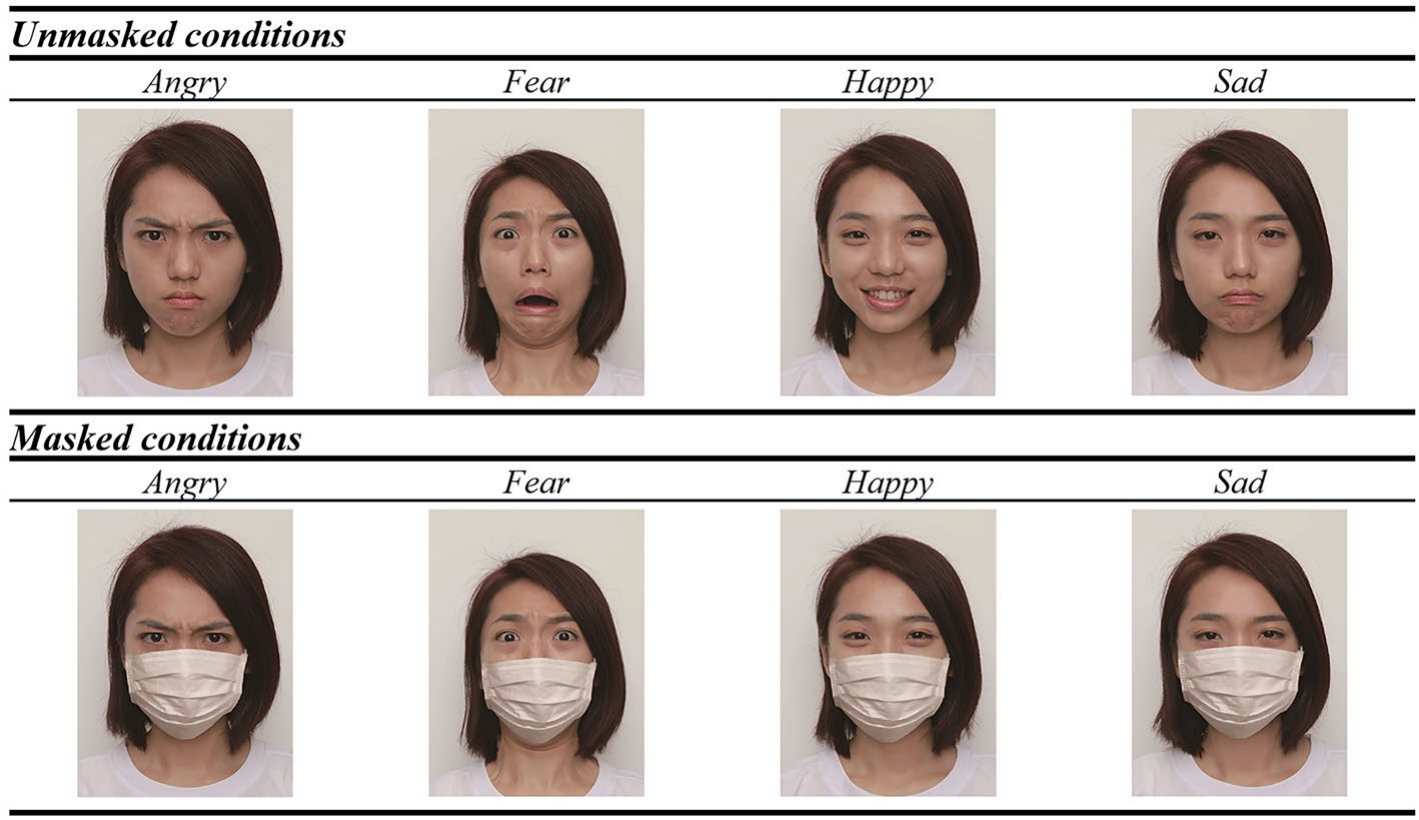
Sample Photos of One Model in Each Experimental Condition

Three hundred twenty colored front-view photos were selected as stimuli from the face database (Chan et al., 2018; Hsiao et al., 2022). The selected photos were taken with 40 Chinese adults (half female, half male). For unmasked stimuli, each adult modeled four emotions (angry, fear, happy, sad) without a face mask. To create masked stimuli, unmasked stimuli were duplicated, and added masks with Adobe Photoshop. Figure 1 illustrates the eight photos that one model contributes to each experimental condition. Angry, fearful, happy, and sad faces were adopted in this study as these four categories of facial expression were found to be commonly perceived in both Chinese and European cultures (Jack et al., 2012, 2016). To standardize the photos, all adults were photographed wearing white T-shirts in front of a white background. All faces subtended a horizontal and vertical visual angle of 6$^\circ$ × 8$^\circ$. Moreover, all photos were scaled and aligned according to the distance between the center of the two eyes. To avoid showing both masked and unmasked versions of the same emotion of a person in the task, two sets of stimuli were created, i.e. Set A and Set B. Each set of stimuli contained 160 photos. In Set A, the first group of 20 models’ masked photos (four emotions per model) were selected, while the second group of 20 models’ unmasked photos were selected. In Set B, however, the first group of 20 models’ unmasked photos were selected, while the second group of 20 models’ masked photos were selected. Only one set of stimuli was used for each participant during the Facial Emotion Recognition Task, with the use of Set A or B counterbalanced across participants. During the task, the set of stimuli was divided into five blocks of 32 trials each. Within each block, trials were randomized, and the number of trials was evenly distributed based on mask and emotion conditions. Moreover, block order was counterbalanced across participants.

#### Self-report questionnaires

**Liebowitz Social Anxiety Scale (LSAS).** Participants’ level of social anxiety was prescreened with the Chinese translation (Chan et al., 2020a) of the Liebowitz Social Anxiety Scale (Liebowitz, 1987). Under 24 social situations, they were to rate their extent of fear (0 = none, 3 = severe) and their frequency of avoidance of the situation (0 = never, 3 = usually) in the past week. The total score ranges from 0 to 144, with higher scores reflecting greater social anxiety levels. LSAS was consistently validated to have good indices of convergence validity and 12-week test–retest reliability (Baker et al., 2002; Fresco et al., 2001). The original (Cronbach α = 0.95; Baker et al., 2002) and Chinese self-reported versions (Cronbach α = 0.97; Chan et al., 2020a) also reported high internal consistency. In the current study, the Cronbach α was 0.96.

**Depression Anxiety Stress Scale-21 (DASS-21).** People with high levels of social anxiety symptoms tended to experience greater depressive symptoms and daily distress (Yoon & Joormann, 2012). The 7-item Depression subscale and the 7-item Stress subscale of the 21-item Depression Anxiety and Stress Scale (Lovibond & Lovibond, 1995) were therefore administered to assess the participants’ subjective levels of depression and stress. A validated Chinese version of DASS-21 (Moussa et al., 2001) was adopted in the study. Participants rated the extent to which the statements applied to them over the past week on a scale from 0 (not at all) to 3 (very much). The raw scores in each subscale were first summed and then doubled to obtain the total scores. Therefore, the total scores on each subscale range from 0 to 42, with a higher score reflecting a greater level of depression or stress. DASS-21 demonstrated great structural validity in the Chinese population, with all subscales possessing good indices of internal consistency and composite reliability (Gong et al., 2010; Moussa et al., 2001). In the current study, the internal consistency of the Depression and Stress subscales were high, with Cronbach α values of 0.82 and 0.83 respectively.

**Demographics.** Age, gender, educational level, normality of vision, and history of mental illnesses were obtained in the self-reported demographic form.

#### Cognitive measures

Executive functions, which are typically negatively associated with social anxiety (Fujii et al., 2013), may also be associated with facial recognition performance and eye movement behaviors (e.g. Circelli et al., 2013; Liu et al., 2025; Zheng & Hsiao, 2023). Therefore, the following tasks were adopted to measure four aspects of executive functions to understand whether social anxiety influences the changes in emotion recognition due to masks even if cognitive abilities were taken into account.

**9-item Raven’s Standard Progressive Matrices (RSPM).** The abbreviated 9-item version of Raven’s Standard Progressive Matrices (Bilker et al., 2012), with a high correlation with the total score in the full set (Raven, 2000), was used to measure participants’ abstract reasoning. Participants were asked to select a missing piece to complete a pattern in a matrix. No time limit was set for the task. The accuracy rate of participants was measured.

**Verbal and Spatial Two-Backs.** Verbal and spatial two-backs were used to measure verbal and visuospatial working memory (Lau et al., 2015). In each trial of the verbal two-back, a single-digit number was shown in the center of the screen for 1000 ms, followed by a blank screen for 2500 ms. Participants needed to judge as accurately and quickly as possible whether the number presented now was the same as the number two trials before. In each trial of the spatial two-back, a symbol would appear in one of the 12 locations for 1000 ms, followed by a blank screen for 2500 ms. Participants needed to judge whether the symbol’s location in the current trial was the same as the location two trials before. Under a viewing distance of 50 cm, each number and symbol subtended a horizontal and vertical visual angle of 1.15$^\circ$ × 1.15$^\circ$. A practice block was given before the participants tried the two test blocks of 26 trials each. The accuracy rate and average reaction time (ms) of correct trials were calculated.

**Flanker Task.** The flanker task was adopted to assess participants’ response inhibition and selective attention (Eriksen & Eriksen, 1974). Participants were asked to judge the direction of the target arrow in the middle as accurately and quickly as possible. In the 40 congruent trials, the target arrow was flanked by four arrows pointing in the same direction as the target arrow. In the 40 incongruent trials, it was flanked by four arrows pointing in the opposite direction to the target arrow. In the 40 neutral trials, it was flanked by four diamond symbols. In each trial, a fixation cross was first presented for 1000 ms, followed by the display of the target arrow and flankers until a response. Under a viewing distance of 75 cm, the target arrow subtended a horizontal and vertical visual angle of 0.69$^\circ$ × 0.69$^\circ$, and each flanker subtended a horizontal and vertical visual angle of 0.92$^\circ$ × 0.92$^\circ$. Participants first familiarized themselves with the task from 12 practice trials and then proceeded to 120 test trials with trial types randomized. The flanker effects in terms of accuracy rate and reaction time (ms) were computed using the formula , in which *I* represented the performance in incongruent trials and *C* represented that in congruent trials.

**Tower of London Task (ToL).** The Tower of London Task was used to assess participants’ ability of executive planning (Berg & Byrd, 2002). In each trial, three color beads were presented in random positions in three rods, with the target positions of the beads presented on the left. Participants were asked to move the bead one at a time to its target position, with the fewest steps possible. Each trial has a time limit of 2 min. One practice trial was given, followed by 12 test trials. Several measures were obtained in each participant: (1) the number of correct trials, (2) the average move count (actual number of moves—minimum number of moves) of correct trials, (3) the average preplanning time (time before initiating the first move) of correct trials (ms), and (4) the average execution time (the total time from the first move to the end move) of correct trials (ms).

### Apparatus

During the Facial Emotion Recognition Task, an EyeLink 1000 eye tracker, placed on a desktop mount, was used to record participants’ eye movements. Participants sat at a viewing distance of 57 cm from the monitor (22-inch, resolution: 1024 × 768 pixels), with their heads placed on a chinrest to stabilize head movements.

The dominant eye of the participants was tracked during the task. A nine-point calibration and validation were performed before each block. Recalibration was also performed when the drift-correction error was greater than the visual angle of 1$^\circ$. For the EyeLink eye tracker, the pupil and corneal reflection tracking mode was used at a sampling rate of 1000 Hz. Also, EyeLink default setting for cognitive research was applied for data collection, i.e. saccade motion threshold of 0.1$^\circ$ of a visual angle, saccade acceleration threshold of 8000 degrees/square second, and saccade velocity threshold of 30$^\circ$.

### Procedures

Participants first filled in an online screening form containing the LSAS one week before they were invited to the lab. During the lab visit, after completing the consent form, they filled in the demographic form. Then, the Facial Emotion Recognition Task was carried out.

During the Facial Emotion Recognition Task, participants first became familiar with the task through a practice block and then proceeded to the five test blocks. In each trial, there was first a solid dot at the center of the screen for drift checks. Once their fixation on the dot had been detected, a photo would appear at one of the four quadrants on the screen until a response was recorded. Participants were asked to identify the emotion of the face presented as accurately and quickly as possible by pressing corresponding keys (*A* for angry, *D* for fear, *H* for happy, *K* for sad). After that, they finished the DASS-21, RSPM, verbal and spatial two-backs, flanker task, and ToL task in order.

### Design

The study was based on a mixed design with SA level as defined by LSAS score as the between-subjects measure, and mask use of the faces (masked, unmasked) and emotion (angry, fear, happy, sad) as the within-subjects measures. During the Facial Emotion Recognition Task, participants identified the four emotions on masked and unmasked faces. The dependent variables were emotion recognition performance (i.e. accuracy rate, false alarm rate, and reaction time of correct trials) as well as their eye movement patterns: the accuracy rate of a target emotion in a condition was computed by ; the false alarm rate of a target emotion in a condition was computed by ; the eye movement patterns of each participant were analyzed using EMHMM and quantified by the A-B scale (see *Eye Movement Data Analysis* for details).

For data analysis, control variables, including demographic information, depressive and stress levels, and cognitive abilities were first examined to see if any differences could be detected between participants with high and low SA, categorized using a median split of LSAS scores: continuous variables were entered into two-tailed independent *t*-tests or Mann–Whitney *U*-tests (if normality is violated under Kolmogorov–Smirnov tests); nominal variables were entered in two-tailed chi-square tests of association. Linear mixed-effect modeling was then used to assess the association between social anxiety level as a *continuum* and facial emotion recognition performance/eye movement behavior in different conditions. More specifically, to predict recognition performance and eye movement pattern, we included LSAS score, mask use, and emotion as the fixed-effect predictors, and participants as random intercepts; control variables having significant differences between high and low SA individuals (gender, DASS Depression and Stress subscores, and ToL preplanning time) were added as covariates; all categorical variables were coded using simple coding. The formulae were specified as follows:
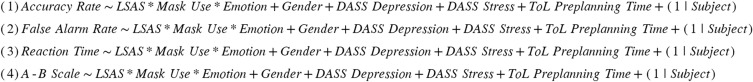


Finally, to examine the relationship between changes in emotion recognition performance and changes in eye movement patterns due to mask use, we quantified the changes in recognition performance and eye movement patterns by each emotion using the mask effect scale = . A more positive value on this scale indicates a relative increase in the particular measure in masked conditions compared to unmasked conditions, while a more negative value indicates a relative decrease. Then, we entered the mask effect scales for recognition performance and eye movement patterns into a two-tailed partial Spearman’s correlation that controlled for all covariates.

### Eye movement data analysis

The eye movement analysis with hidden Markov model (EMHMM) toolbox in MATLAB (Version 0.80) was used to analyze the eye movement data (Chuk et al., 2014; available at http://visal.cs.cityu.edu.hk/research/emhmm/). A hidden Markov model was used to summarize the eye movement patterns in each experimental condition, i.e. mask (masked, unmasked) x emotion (angry, fear, happy, sad), for each participant. The variational Bayesian expectation–maximization algorithm (Bishop, 2006) was used to train each hidden Markov model, and the optimal number of ROIs for each individual model was determined from a preset range of 1 to 11. Using the variational hierarchical expectation–maximization algorithm (Coviello et al., 2014), all individual hidden Markov models were clustered into two representative patterns during emotion recognition, named Pattern A and Pattern B. For each participant in each condition, we then quantify the eye movement pattern using the A–B scale (e.g., Chan et al., 2018, 2020a, 2020b; Hsiao et al., 2022; Zheng & Hsiao, 2023): . In the formula,* a* represents the log-likelihood of the eye movement data generated by Pattern A, while* b* represents the log-likelihood of the eye movement data generated by Pattern B. Thus, a more positive value on the A-B scale indicates a greater similarity of using Pattern A, whereas a more negative value on the A-B scale indicates a greater similarity of using Pattern B.

### Transparency and openness

We report how we determined our sample size, all manipulations, and all measures in the study. Data were analyzed using Jamovi (Version 2.3.21) and R Statistical Language (Version 4.1.0). The GAMLj package in Jamovi was used to calculate the p-values in mixed-effect models based on Satterthwaite’s approximation for degrees of freedom. All materials, data, and analysis code can be found on the Open Science Framework at https://osf.io/ypcqh/. This study’s design and its analysis were not pre-registered.

## Results

The descriptive statistics of demographic, questionnaire, and cognitive measures are summarized in Table 1.

**Table 1:** Summary of Demographic, Questionnaire and Cognitive Characteristics

	High SA (n = 44)			Low SA (n = 44)				
	*M*	*SD*		*M*	*SD*	*U*	*p*	*r*_*rb*_
*Demographics*								
*Age (years)*	21.25	1.48		21.09	0.71	929.00	.708	.04

	*Counts*	*%*		*Counts*	*%*	*χ*^*2*^	*p*	*V*
*Gender*						6.90	.009**	.28
Female	33	75		21	47.73			
Male	11	25		23	52.27			
*Education*						2.00	.736	.15
Year 1	1	2.27		0	0			
Year 2	3	6.82		3	6.82			
Year 3	10	22.73		10	22.73			
Year 4	30	68.18		30	68.18			
Year 5	0	0		1	2.27			

	*M*	*SD*		*M*	*SD*	*t/U*	*p*	*d/r*_*rb*_
*Questionnaires*								
*LSAS*	53.93	12.49		20.84	8.62	14.46	<.001***	3.08
*DASS-21*								
Depression Subscale	11.18	8.05		6.05	4.93	3.61	<.001***	0.77
Stress Subscale	14.64	7.57		8.36	6.80	4.09	<.001***	0.87
*Cognitive tasks*								
*RSPM ACC*^*#*^	.81	.14		.84	.14	852.00	.319	.12
*Two-backs*								
Spatial ACC^*#*^	.92	.07		.92	.10	924.50	.717	.04
Spatial RT (ms)	1046.36	240.06		1086.15	198.44	− 0.85	.399	− 0.18
Verbal ACC^*#*^	.92	.06		.93	.06	858.50	.358	.11
Verbal RT (ms)^*#*^	811.33	187.87		838.08	192.38	871.00	.423	.10
*Flanker effect*								
ACC^*#*^	−.02	.02		−.02	.02	931.00	.755	.04
RT	.08	.03		.07	.03	1.68	.096	0.36
*Tower of London*								
Correct trial no.^*#*^	11.61	1.35		11.89	0.32	938.50	.673	.03
Move count	1.04	0.91		1.00	0.94	0.18	.855	0.04
Preplanning time (ms)	7841.50	3676.04		9982.37	5288.59	− 2.20	.030*	− 0.47
Execution time (ms)	10,169.36	4423.81		10,104.66	4480.19	0.07	.946	0.01

LSAS, Liebowitz Social Anxiety Scale; DASS-21, 21-item Depression Anxiety Stress Scale; RSPM, 9-item Raven’s Standard Progressive Matrices; ACC, Accuracy rate; RT, reaction time of correct trials; *r*_rb_, rank-biserial correlation coefficient

*p <.05, ** *p* <.01, *** *p* <.001

^#^indicates the variables that used Mann–Whitney *U* tests and rank-biserial correlation coefficients

Significant differences between high and low SA participants were detected in the following control variables: gender, DASS-21 Depression and Stress subscores, and preplanning time in the Tower of London task. First, there were significantly more females with high SA than low SA, *χ*^*2*^ (1) = 6.90, *p* = 0.009, *V* = 0.28, 95% confidence interval (CI) [0.13, 0.50]. Second, high SA participants had significantly higher DASS-21 Depression subscores, Welch’s* t* (71.25) = 3.61, *p* < 0.001, *d* = 0.77, [0.32, 1.21]. Third, significantly higher DASS-21 Stress subscores were also observed in high SA participants, *t* (86) = 4.09, *p* < 0.001, *d* = 0.87, [0.41, 1.32]. And fourth, high SA participants used significantly less preplanning time during the Tower of London task, Welch’s *t* (76.69) = − 2.20, *p* = 0.030, *d* = − 0.47, [− 0.90, − 0.04]. These four variables were added as covariates and controlled for in subsequent analyses.

### Facial emotion recognition performance

**Fig. 2:**
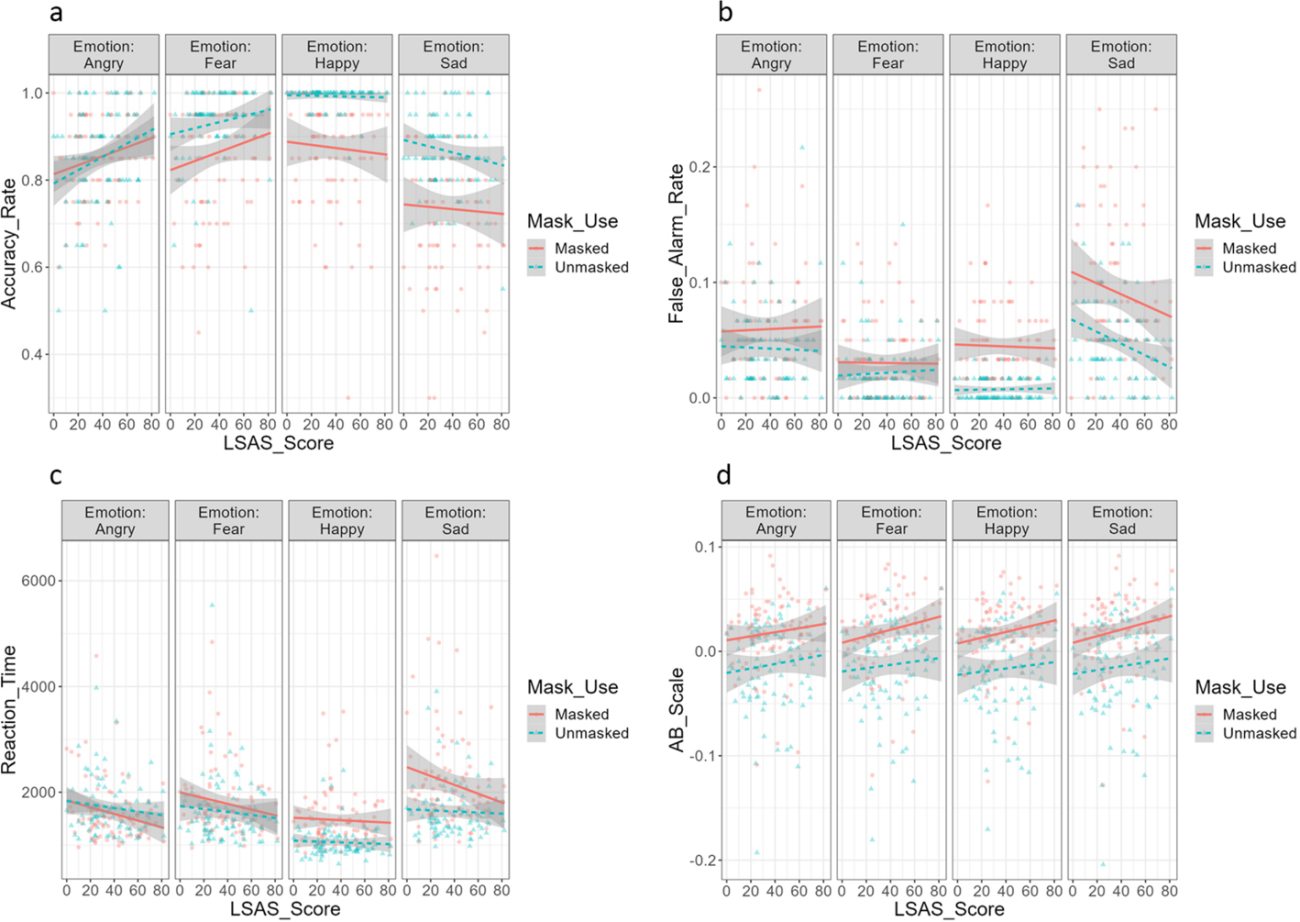
Association Between LSAS and Facial Emotion Recognition Performance. *Note.* **a** Accuracy rate. **b** False alarm rate. **c** Reaction time. **d** A-B Scale. A more positive value indicates a more eye-centered strategy; a more negative value indicates a more nose-centered strategy

The summary of facial emotion recognition performance is described in Table 2 and visualized in Fig. 2.

**Table 2:** Summary of recognition performance and A–B scales in facial emotion recognition task

	Angry faces		Fearful faces		Happy faces		Sad faces	
	Unmasked	Masked	Unmasked	Masked	Unmasked	Masked	Unmasked	Masked
*Accuracy rate*								
High SA	.87 (.12)	.86 (.09)	.94 (.08)	.88 (.10)	.99 (.03)	.86 (.14)	.86 (.10)	.75 (.12)
Low SA	.83 (.11)	.84 (.10)	.92 (.09)	.84 (.14)	.99 (.02)	.89 (.11)	.87 (.07)	.72 (.15)
*False alarm rate*								
High SA	.04 (.04)	.06 (.04)	.02 (.03)	.03 (.03)	.01 (.01)	.04 (.03)	.04 (.03)	.08 (.06)
Low SA	.05 (.03)	.06 (.06)	.02 (.03)	.03 (.03)	.01 (.01)	.05 (.04)	.05 (.04)	.10 (.07)
*Reaction time (ms)*								
High SA	1619.04 (433.52)	1506.51 (436.55)	1558.37 (458.45)	1672.38 (441.14)	1032.58 (282.60)	1441.39 (493.84)	1623.55 (440.36)	1997.97 (702.28)
Low SA	1800.80 (572.19)	1706.57 (674.29)	1709.93 (754.54)	1917.51 (775.62)	1074.56 (306.57)	1507.16 (524.90)	1653.63 (592.35)	2326.26 (1090.23)
*A-B scale*								
High SA	−.01 (.04)	.02 (.04)	−.01 (.04)	.03 (.04)	−.01 (.04)	.02 (.03)	−.01 (.04)	.03 (.04)
Low SA	−.02 (.04)	.01 (.03)	−.02 (.04)	.01 (.03)	−.02 (.04)	.01 (.03)	−.02 (.05)	.01 (.03)

MM.MM indicates mean. (SD.SD) indicates standard deviation. A more positive value on the A–B scale indicates a more eye-centered strategy; a more negative value on the A–B scale indicates a more nose-centered strategy

#### Accuracy rate

First, a significant main effect of LSAS showed that higher LSAS scores were associated with higher accuracy rates in general, *F* (1, 684) = 4.74, *p* = 0.030, *n*^*2*^_*p*_ = 0.01, 90% CI [0.0004, 0.0209].^1^ A significant interaction between LSAS and emotion further revealed that the higher accuracy rate associated with social anxiety was emotion-specific, *F* (3, 684) = 4.62, *p* = 0.003, *n*^*2*^_*p*_ = 0.02, [0.004, 0.037]. Specifically, the positive association between LSAS and accuracy rates was only shown for angry, *F* (1, 509.53) = 11.88, *p* < 0.001, *n*^*2*^_*p*_ = 0.02, [0.01, 0.05], and fearful faces, *F* (1, 509.53) = 5.97, *p* = 0.015, *n*^*2*^_*p*_ = 0.01, [0.001, 0.032], but not for happy, *p* = 0.856, *n*^*2*^_*p*_ = 0.00, [0.00, 0.00], or sad faces, *p* = 0.390, *n*^*2*^_*p*_ = 0.00, [0.00, 0.01]. Second, a significant main effect of mask use was observed, *F* (1, 684) = 103.07, *p* < 0.001, *n*^*2*^_*p*_ = 0.13, [0.09, 0.17]. Participants generally identified facial emotions less correctly in masked conditions. There was also a significant interaction between mask use and emotion, *F* (3, 684) = 15.23, *p* < 0.001, *n*^*2*^_*p*_ = 0.06, [0.03, 0.09]. A post-hoc test with Bonferroni correction showed that the negative impact of mask use on accuracy rate was only shown in fearful, happy, and sad faces, *p*s < 0.001, while angry faces had a similar accuracy rate in both the masked and unmasked conditions, *p* = 1.000. This suggested that participants mainly relied on the regions around the eyes to identify angry faces while recognizing other emotions required information in the lower face. Thirdly, a significant main effect of emotion was also revealed, *F* (3, 684) = 55.35, *p* < 0.001, *n*^*2*^_*p*_ = 0.20, [0.15, 0.24], and the Bonferroni-corrected post-hoc test showed that accuracy rates significantly differed among four emotions, with the highest accuracy rates for happiness, then fear, followed by anger, and lastly sadness (happiness vs. fear: *p* = 0.005; other pairwise comparisons: *ps* < 0.001). Contrary to our hypothesis, there was neither a significant interaction between LSAS and mask use, *F* (1, 684) = 0.00, *p* = 0.998, *n*^*2*^_*p*_ = 0.00, [0.00, 0.00], nor a LSAS x mask use x emotion interaction, *F* (3, 684) = 0.35, *p* = 0.789, *n*^*2*^_*p*_ = 0.00, [0.00, 0.01].

#### False alarm rate

Despite a non-significant main effect of LSAS, *F* (1, 684) = 3.84, *p* = 0.050, *n*^*2*^_*p*_ = 0.01, [0.00, 0.02], there was a significant interaction between LSAS and emotion, revealing an emotion-specific association between social anxiety and false alarm rate, *F* (3, 684) = 2.92, *p* = 0.033, *n*^*2*^_*p*_ = 0.01, [0.001, 0.027]. Specifically, higher LSAS scores were significantly associated with lower false alarm rates only for sad faces, *F* (1, 509.53) = 12.58, *p* < 0.001, *n*^*2*^_*p*_ = 0.02, [0.01, 0.05], but not for angry, *p* = 0.772, *n*^*2*^_*p*_ = 0.00, [0.00, 0.01], fearful, *p* = 0.880, *n*^*2*^_*p*_ = 0.00, [0.00, 0.00], or happy faces, *p* = 0.705, *n*^*2*^_*p*_ = 0.00, [0.00, 0.01]. In addition, a significant main effect of mask use indicated that participants overall had higher false alarm rates during masked conditions, *F* (1, 684) = 83.57, *p* < 0.001, *n*^*2*^_*p*_ = 0.11, [0.07, 0.15]. There was also a significant interaction between mask use and emotion, *F* (3, 684) = 7.93, *p* < 0.001, *n*^*2*^_*p*_ = 0.03, [0.01, 0.06]. A post-hoc test with Bonferroni correction showed that the increase in false alarm rate due to mask use was only shown in happy and sad faces, *p*s < 0.001, but not in angry, *p* = 0.110, or fearful faces, *p* = 1.000. Finally, a significant main effect of emotion was observed,* F* (3, 684) = 55.16, *p* < 0.001, *n*^*2*^_*p*_ = 0.19, [0.15, 0.24]. The Bonferroni-corrected post-hoc test showed that participants misidentified other target emotions as sadness the most, followed by anger, and finally happiness as well as fear the least, with the false alarm rates for the latter two emotions being roughly the same (happiness vs fear: *p* = 1.000; other pairwise comparisons: *ps* < 0.001). Contrary to our hypothesis, there was neither a significant interaction between LSAS and mask use, *F* (1, 684) = 0.00, *p* = 0.999, *n*^*2*^_*p*_ = 0.00, [0.00, 0.00] nor a LSAS x mask use x emotion interaction, *F* (3, 684) = 0.08, *p* = 0.969, *n*^*2*^_*p*_ = 0.00, [0.00, 0.00].

#### Reaction time

First, a significant main effect of mask use was found: participants tended to have longer reaction times when identifying facial emotions in masked conditions, *F* (1, 602) = 76.81, *p* < 0.001, *n*^*2*^_*p*_ = 0.11, [0.08, 0.15]. There was no main effect of LSAS, *F* (1, 82) = 0.01, *p* = 0.937, *n*^*2*^_*p*_ = 0.00, [0.00, 0.01]. A significant interaction between LSAS and mask use was observed, *F* (1, 602) = 5.20, *p* = 0.023, *n*^*2*^_*p*_ = 0.01, [0.001, 0.025]; however, in contrast to our hypothesis, the increase in reaction time due to mask use was smaller among participants with higher LSAS (+ 1 *SD*), *Β* = 185.22, 95% CI [105.83, 264.60], *t* (602) = 4.58, *p* < 0.001, compared with those with lower LSAS (− 1 *SD*), *Β* = 315.61, [236.22, 394.99], *t* (602) = 7.81, *p* < 0.001, suggesting that social anxiety was associated with *less* impairment in reaction time due to mask use. Second, there was a significant main effect of emotion, *F* (3, 602) = 87.78, *p* < 0.001, *n*^*2*^_*p*_ = 0.30, [0.25, 0.35]. The post-hoc test with Bonferroni correction showed that participants correctly identified happiness the fastest, followed by anger and fear with similar reaction times, and sadness the slowest (anger vs fear: *p* = 0.983; other pairwise comparisons: *ps* < 0.001). Moreover, there was a significant interaction between emotion and mask use, *F* (3, 602) = 24.17, *p* < 0.001, *n*^*2*^_*p*_ = 0.11, [0.07, 0.14]. A post-hoc test with Bonferroni correction showed that the increase in reaction time due to mask use was only shown in happy and sad faces, *p*s < 0.001, but not in angry, *p* = 1.000, or fearful faces, *p* = 0.141. Finally, there was neither a significant interaction between LSAS and emotion, *F* (3, 602) = 1.54, *p* = 0.203, *n*^*2*^_*p*_ = 0.01, [0.00, 0.02] nor a LSAS x mask use x emotion interaction, *F* (3, 602) = 1.01, *p* = 0.387, *n*^*2*^_*p*_ = 0.01, [0.00, 0.01].

### Eye movement patterns during facial emotion recognition

**Fig. 3:**
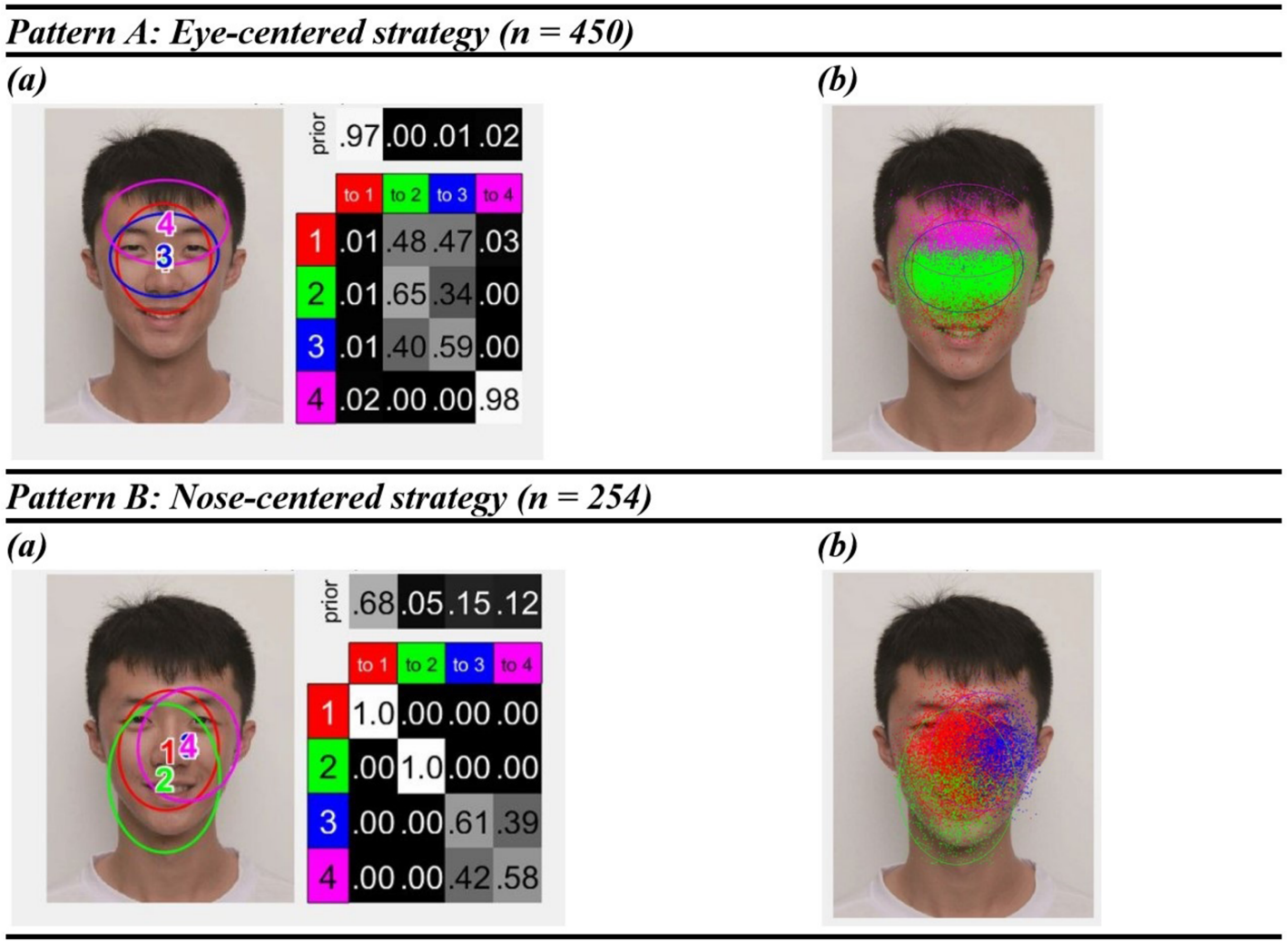
The Two Representative Patterns Found in EMHMM. *Notes.* **a** The representative hidden Markov model. The ellipses on the left show the ROIs as 2-D Gaussian emissions. The table on the right shows the priors (the probabilities that a fixation sequence starts from the ellipses) and the transition probabilities from one ROI to another in the sequence. **b** The actual assignment of fixations to each ROI

The two representative eye movement patterns (Pattern A and Pattern B) during facial emotion recognition found by clustering in EMHMM are shown in Fig. 3. The two patterns were significantly different, as the data from participants adopting Pattern A were more likely to be generated by Pattern A HMM than Pattern B HMM, *t* (449) = 14.49, *p* < 0.001, *d* = 0.68, [0.58, 0.79], and vice versa for data from participants adopting Pattern B (following Chuk et al., 2014), *t* (253) = 9.68, *p* < 0.001, *d* = 0.61, [0.47, 0.74]. In Pattern A, a scan path mostly started with a fixation at a broad region that centered at the midpoint between two eyes and spread from the eyebrows and above the upper lip (*red*, 97%), with small occasions to start at the forehead region (*magenta*, 2%) or at a smaller region only covering the eye and the nose regions (*blue*, 1%). After the fixation to the broad region (*red*), the path typically switched to narrower regions covering the nose and eye regions (*red to blue*, 47%) or eye region only (*red to green*, 48%), and then mostly remained at the same regions. In contrast, in Pattern B, the scan path typically started at a region that centered at the nose tip and spread from the brow ridge to the mouth region (*red*, 68%), with probabilities of starting at a smaller region centered at the left cheek of the model (*blue*, 15%; *magenta*, 12%) or small occasions that start at a broad region that centered the mouth (green, 5%). Participants tended to remain at the same fixation region after making their first fixation to the nose-tip-centered region (*red to red*, 100%). In sum, Pattern A represented a more eye-centered viewing strategy when identifying facial emotions, whereas Pattern B described a more nose-centered strategy. Thus, we termed Pattern A the “eye-centered strategy” and Pattern B the “nose-centered strategy”. Afterward, we examined whether social anxiety was associated with the differences in the tendency to use a more eye-centered or a nose-centered strategy across conditions (quantified by the A–B scale).

The mixed-effect model showed a significant main effect of mask use, *F* (1, 602) = 1089.52, *p* < 0.001, *n*^*2*^_*p*_ = 0.64, [0.61, 0.67], with an overall increase in using eye-centered strategy associated with mask use. There was no significant main effect of LSAS, *F* (1, 82) = 0.32, *p* = 0.570, *n*^*2*^_*p*_ = 0.00, [0.00, 0.06], or emotion, *F* (3, 602) = 1.41, *p* = 0.240, *n*^*2*^_*p*_ = 0.01, [0.00, 0.02]. There was also no significant interaction effect: the interaction between LSAS and mask use, *F* (1, 602) = 3.47, *p* = 0.063, *n*^*2*^_*p*_ = 0.01, [0.00, 0.02]; the emotion x LSAS interaction, *F* (3, 602) = 0.17, *p* = 0.918, *n*^*2*^_*p*_ = 0.00, [0.00, 0.00]; the emotion x mask use interaction, *F* (3, 602) = 1.00, *p* = 0.391, *n*^*2*^_*p*_ = 0.00, [0.00, 0.01]; the interaction between emotion, mask use, and LSAS, *F* (3, 602) = 0.59, *p* = 0.619, *n*^*2*^_*p*_ = 0.00, [0.00, 0.01]. This indicated that participants with different social anxiety levels adopted a similar eye movement strategy during emotion recognition across emotion and mask use conditions (see *Supplementary Materials B* for a comparison with results using a predefined ROI approach).

### Association between performance changes and eye movement changes due to mask

Partial Spearman correlation analyses controlling for gender, DASS-21 Depression and Stress subscores, and ToL preplanning time were performed on participants’ mask effect scales of the emotion recognition performance and eye movement patterns by emotions, which showed no significant correlations between any recognition performance changes and A–B scale changes due to mask use, *p*s > 0.1 (see *Supplementary Materials C*). This suggests, in the whole sample, the extent of the worsening emotion recognition performance (i.e. reduction in accuracy rate, rise in false alarm rate, and increase in reaction time) did not have a significant linear relationship with the extent to which participants switched to a more eye-centered strategy.

**Fig. 4:**
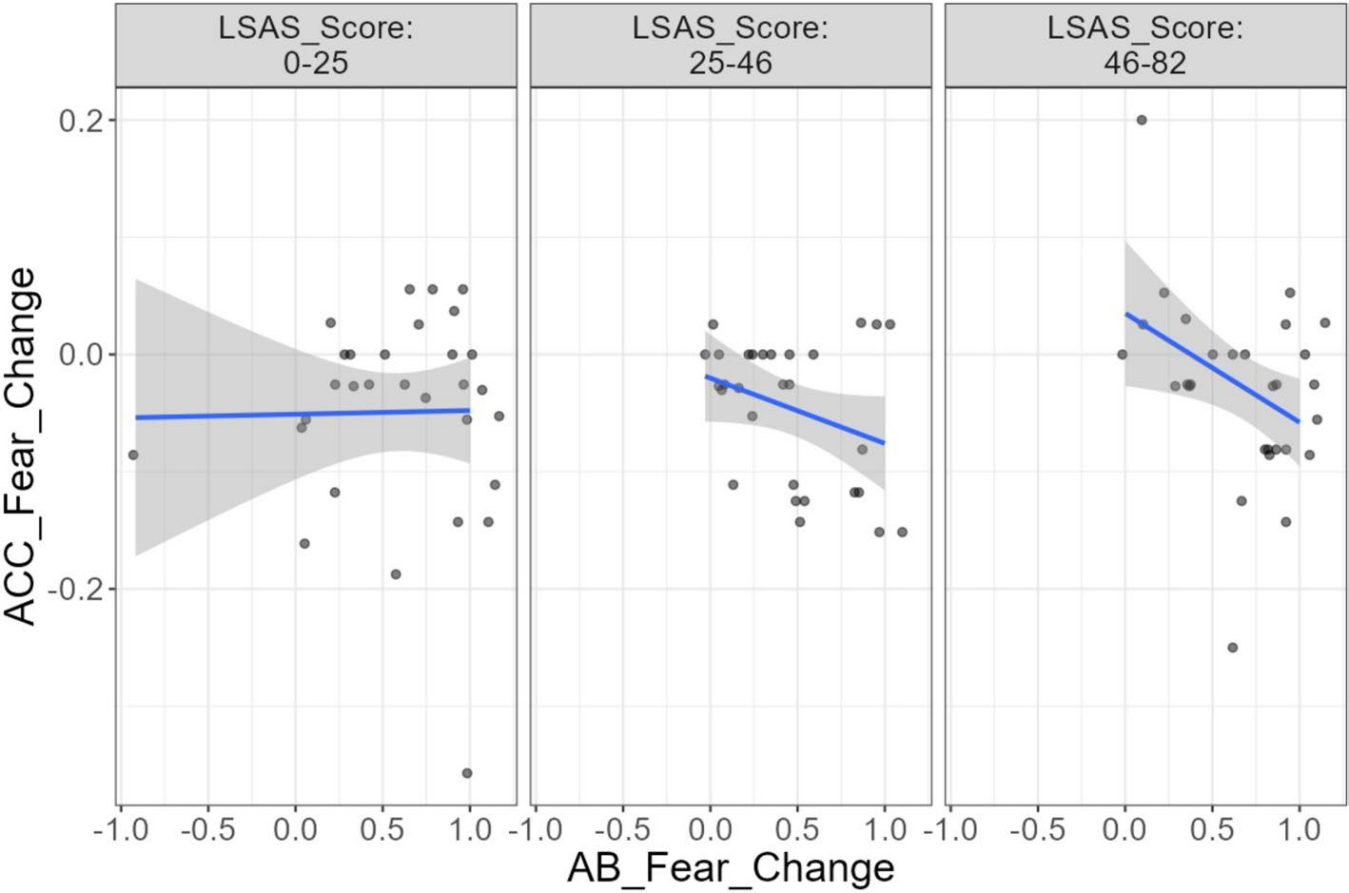
Association Between A-B Scale Changes and Accuracy Rate Changes for Fearful Faces. *Notes.* A more positive value in AB_Fear_Change indicates a larger increase in switching to the eye-centered strategy when recognizing fearful faces due to mask use. A more negative value in ACC_Fear_Change indicates a larger decrease in accuracy rate for fearful faces due to mask use

Since previous research has reported an association between recognition performance change and eye movement pattern change due to masks in the non-clinical population (e.g., Hsiao et al., 2022; Zheng et al., 2023), and we have already found a close link between higher social anxiety and enhanced emotion recognition in terms of accuracy and false alarm rates, we further examined whether social anxiety would moderate the relationship between A–B scale changes and facial emotion recognition performance changes due to masks. This examination could provide crucial information on how changes in attentional strategies due to masks potentially affect the heightened emotion sensitivity associated with subclinical social anxiety. Thus, we ran *exploratory* moderation analyses of SA levels on the association between the changes in recognition performance and changes in eye movement patterns while controlling for all covariates, using the ordinary least squares regression model in the GAMLj package in Jamovi. For each emotion, (1) A–B scale changes, (2) LSAS, (3) the interaction between LSAS and A–B scale changes, and (4–7) four covariates were added as the predictors, while the three recognition performance changes were each set as the outcome variable. Only the regression model with accuracy rate changes for fearful faces as the outcome accounted for a significant portion of variance, *F* (7, 80) = 2.21, *p* = 0.042, *R*^*2*^ = 0.16, 90% CI [0.003, 0.236]. The interaction between A–B scale changes and LSAS significantly predicted accuracy rate changes for fearful faces, *β* = − 0.34,* p* = 0.008, suggesting that SA levels served as a moderator for the association between changes in eye movement patterns and changes in accuracy rate for fearful faces. Simple slope tests further revealed that the larger extent of switching to the more eye-centered strategy for fearful faces was significantly associated with a larger drop in accuracy rate among individuals with higher (+ 1 *SD*) LSAS, *β* = − 0.46, *p* = 0.004. In contrast, it was non-significantly associated with a smaller drop in accuracy rate among those with lower (− 1 *SD*) LSAS, *β* = 0.22, *p* = 0.212 (see Fig. 4).

## Discussion

The purpose of this study was to investigate the performance and eye movement patterns during facial emotion recognition among individuals with subclinical levels of social anxiety, in particular when the faces were covered by surgical masks.

### Emotion recognition performance

Our collective results showed individuals with higher social anxiety did not suffer a greater impairment in recognizing masked facial expressions when controlling for gender, depressive and stress levels, and executive planning ability. In our main analysis, we failed to find significant interaction effects between social anxiety level and mask use on accuracy rate and false alarm rate. Moreover, we found participants with higher social anxiety instead showed a smaller increase in reaction times due to mask use. Therefore, our results did not support our hypothesis that the emotion recognition performance of individuals with higher social anxiety would be more impaired under masked conditions. In contrast, we observed that social anxiety was associated with higher accuracy rates for angry and fearful faces and lower false alarm rates for sad faces regardless of mask use.

Some previous studies have suggested social anxiety is associated with interpretation bias, leading to higher false alarms of negative emotions in recognizing ambiguous facial expressions (Bell et al., 2011; Heuer et al., 2010; Maoz et al., 2016; Prieto-Fidalgo et al., 2023). In contrast, our findings using a subclinical sample showed that social anxiety was associated with lower false alarm rates for sad faces regardless of mask use. Moreover, our results in accuracy rate are inconsistent with the previous findings from RMET tests that social anxiety is associated with specific deficits in inferring mental states from the eyes in clinical samples (Hezel & McNally, 2014; Maleki et al., 2020; Öztürk et al., 2022; Washburn et al., 2016). Our results showed that higher social anxiety was associated with higher accuracy rates of recognizing angry and fearful faces regardless of mask conditions. Along with a few studies using subclinical samples (Sutterby et al., 2012; Tibi-Elhanany & Shamay-Tsoory, 2011), the current results indicate that social anxiety, at subclinical levels, may rather be associated with an *enhanced* theory of mind, especially for negative emotions. When people with subclinical levels of social anxiety are more sensitive to others’ negative evaluations reflected by facial expressions, they may feel more self-conscious of their behavior during social interactions, causing more anxiety. On the contrary, SAD patients may suffer from an impairment in decoding social signals and experience difficulties in how to respond to them. Indeed, Nikolić and colleagues (2019) found a quadratic pattern of RMET performance and SA levels in children: at subclinical levels, social anxiety was associated with better RMET performance, which was mediated by blushing, a physiological marker of heightened self-consciousness. At clinical levels, however, social anxiety was associated with worse RMET performance. Future research should note the potential difference in ToM mechanisms underlying social anxiety in the clinical and subclinical populations, and examine it with other paradigms.

### Eye movement patterns

In addition, our result was also inconsistent with our hypothesis regarding eye movement patterns. We did not find a significant interaction effect between social anxiety levels and mask use, nor did we find a main effect of SA levels on eye movement patterns, suggesting that social anxiety was not associated with a particular eye movement strategy. Therefore, our result is inconsistent with previous studies indicating that high SA individuals tended to avoid direct eye gaze compared with low SA counterparts (Horley et al., 2003, 2004; Moukheiber et al., 2010, 2012; Staugaard & Rosenberg, 2011; Weeks et al., 2013, 2019). The heterogeneous attentional styles within the socially anxious population may explain our failure to uncover the singular eye movement pattern linked to social anxiety. Recent research suggests there may be heterogeneity of threat-related attentional processes among people with high levels of social anxiety (Chan et al., 2020a; Evans et al., 2020; Rubin et al., 2024). In particular, F. H. F. Chan, Barry, and colleagues (2020) used EMHMM to evaluate the eye movement patterns of healthy college students during free-viewing of neutral and angry faces. Those who consistently adopted either the eye-centered strategy (representing attentional vigilance towards threats) or the nose-centered strategy (representing attentional avoidance), when viewing *both* facial expressions tended to have higher levels of social anxiety. Hence, two distinct threat-related attentional biases—vigilance and avoidance—may exist among high SA individuals, which may mitigate our efforts to find a uniform eye movement pattern associated with social anxiety in the present study.

Interestingly, our exploratory analysis found the relationship between changes in accuracy rates for fearful faces due to mask use and changes in eye movement patterns was moderated by social anxiety level. For participants with higher social anxiety, the increase in using the eye-centered strategy was significantly associated with a larger drop in accuracy rate for fearful faces. For those with lower social anxiety, however, the increase in using the eye-centered strategy was non-significantly associated with a smaller drop in accuracy rate for fearful faces. Our findings indicate that, although individuals with higher levels of subclinical social anxiety possess a generally heightened sensitivity to negative emotions, their sensitivity is affected by their changes in face-viewing strategy in response to mask use: if they directed their attention more to the eye region as the result of mask use, they performed *worse*, in contrast to individuals with lower levels of social anxiety. On top of that, the non-significant interaction effects of SA levels and mask use on emotion recognition performance highlights that the presence of masks *by itself* does not lead to greater emotion recognition impairment in subclinical SA individuals, but only when they choose to change their viewing strategies in light of the masks.

This exploratory finding is in line with related findings in alexithymia. Alexithymia, the impaired ability to describe one’s own feelings, is positively related to social anxiety (Dalbudak et al., 2013; Panayiotou et al., 2020). In a recent study, Fujiwara (2018) found that for people with high alexithymia, more attention to the eyes was significantly related to lower decoding accuracy of facial emotions; whereas for people with low alexithymia, more attention to the eyes was related to better decoding accuracy at a trend level.

The exact mechanism behind this finding requires further research. However, one possible reason may be that individuals with higher social anxiety feel more threatened when looking at the eyes directly. As mentioned, high SA individuals experienced heightened physiological responses (Myllyneva et al., 2015; Wieser et al., 2009) and displayed higher neural activation in the fear circuitry (Schneier et al., 2009) viewing direct eye gaze. As the eyes on mask faces are more salient, they may experience even more distress when adopting a more eye-centered strategy, which may lead to more impairment in emotion recognition.

Together our findings hold important implications not only for the theoretical accounts of subclinical social anxiety but also for the evidence-based practice in mitigating sociocognitive challenges faced by anxious individuals. First, through manipulating mask use, we found that the presence of masks *by itself* is not likely to make significant impairments in facial emotion recognition in individuals with subclinical social anxiety. Instead, subclinical social anxiety is associated with heightened sensitivity to negative facial emotions regardless of mask use, suggesting that subclinical social anxiety may rather be related to an advanced ToM ability. More importantly, through the machine-learning-based method EMHMM, our study provides new insight into how such heightened sensitivity associated with subclinical social anxiety is influenced by attention to the eyes. This finding supports the theoretical proposals regarding the interplay between attention and interpretation processes in the development of anxiety (i.e. the combined cognitive bias hypothesis, Hirsch et al., 2006; for a recent review, see Leung et al., 2022). Providing interpretation training alone is, therefore, insufficient and likely ineffective for individuals with subclinical social anxiety to mitigate the impact of mask use on facial emotion processing. Instead, future research should explore the effectiveness of combining eye contact training (Matsumoto et al., 2023) in reducing distress and its influence on facial emotion sensitivity among socially anxious individuals, which may offer a potential avenue for enhancing interventions for social anxiety.

### Limitations

Some limitations of the study should be noted. First, our study excluded people who reported any past or current history of psychiatric illnesses, including social anxiety disorder. Therefore, we were unable to examine whether similar effects would generalize to SAD patients. Second, we failed to match the gender ratio between high and low SA participants. Although we statistically controlled for the effect of gender in our analyses, our results might not rule out the possible gender difference. Third, readers should be cautious of the results that were just below the 0.05 threshold of statistical significance. More studies should be done to examine the enhanced facial emotion sensitivity in subclinical social anxiety, and whether looking to the eyes interferes with such sensitivity. Finally, the use of static faces as stimuli may reduce the ecological validity of findings. In real life, facial expressions are dynamic in nature. There is some evidence that SAD patients had an advantage in recognizing anger from static faces but not from dynamic faces (Torro-Alves et al., 2016). Thus, we cannot eliminate the possibility that the better recognition performance of participants with higher social anxiety in our study may be confounded by static faces. Future research is needed to replicate the present findings in a more naturalistic setting, such as producing true-to-life clips of dynamic facial expressions with professional actors (Leitner et al., 2022).

## Conclusion

The present study examined the effect of surgical masks on facial emotion recognition and eye movement patterns of people with subclinical social anxiety in the general population. Results suggest that people with higher levels of social anxiety did not suffer larger impairment in facial emotion recognition due to the presence of masks, compared with those with lower social anxiety levels. Instead, higher social anxiety levels were associated with higher accuracy rates for angry and fearful faces and lower false alarm rates for sad faces. Furthermore, eye movement analysis with hidden Markov models did not find evidence of eye gaze avoidance. However, exploratory analysis indicates that the increase in eye-centered strategy due to masks was related to a larger drop in accuracy rates for fearful faces among people with higher levels of social anxiety. Our collective findings suggest that social anxiety, at least subclinically, may be associated with a generally heightened sensitivity to negative facial emotions. However, such heightened sensitivity diminishes if they switch to a more eye-centered strategy due to mask use.

## Supplementary Information

Supplementary material 1

## Data Availability

Materials, data, and analysis code in the current study are available in the Open Science Framework repository, https://osf.io/ypcqh/. This study was not pre-registered.

## Abbreviations

ACC: Accuracy rate
DASS-21: 21-Item Depression Anxiety Stress Scale
EMHMM: Eye movement analysis with hidden Markov models
FA: False alarm rate
HMM: Hidden Markov model
LSAS: Liebowitz Social Anxiety Scale
RMET: Reading the Mind in the Eyes Test
ROIs: Regions of interest
RSPM: 9-Item Raven’s Standard Progressive Matrices
RT: Reaction time of the correct trials
SA: Social anxiety
SAD: Social anxiety disorder
ToL: Tower of London task
ToM: Theory of mind

## Footnotes

1 Ninety-percent CI instead of 95% CI is reported for *F* tests as *F* tests are one sided (Steiger, 2004).

## Declarations

### Open practices statement

All materials, data, and analysis code can be found on the Open Science Framework at https://osf.io/ypcqh/. This study’s design and its analysis were not pre-registered.
