# Supplementary material for: Impact of mask use on facial emotion recognition in individuals with subclinical social anxiety: an eye-tracking study
Source: Cogn Res Princ Implic. 2025 Jun 2;10:25. doi: 10.1186/s41235-025-00635-4 (PMC12130428; doi:10.1186/s41235-025-00635-4)
Supplement: Supplementary file 1 — Supplementary material 1 [file 41235_2025_635_MOESM1_ESM.doc]

# Supplementary Materials

## A. Facial Emotion Sensitivity Analysis

Given the consistent results in accuracy and false alarm rates showing that higher social anxiety levels were associated with better facial emotion recognition, here we incorporated the two measures by replacing the outcome variable in our mixed-effect model with the sensitivity index *d*’[[1]](#footnote-2) (Figure 1). The main effects of LSAS, mask use, and emotion remained significant after controlling for gender, DASS-21 Depression and Stress subscores, and ToL preplanning time. First, higher LSAS scores were significantly linked with higher *d*’, *F* (1, 82) = 5.54, *p* = .021, *n^2^_p_* = .06, 90% CI [.01, .16]. Second, mask use significantly reduced *d*’, *F* (1, 602) = 428.62, *p* < .001, *n^2^_p_* = .42, [.37, .46]. Third, a significant main effect of emotion was also revealed, *F* (3, 602) = 275.41, *p* <.001, *n^2^_p_* = .58, [.54, .61]. A post-hoc test with Bonferroni correction showed that sensitivity significantly differed among four emotions, with happiness having the highest *d*’, followed by fear, and then anger, and finally sadness, *ps* < .001. Consistent with our main analysis, we also found a significant interaction between LSAS scores and emotion, *F* (3, 602) = 3.76, *p* = .011, *n^2^_p_* = .02, [.002, .036]. However, here we found that higher LSAS scores were only significantly linked to higher *d*’ for angry, *p* <.001, *n^2^_p_* = .05, [.01, .10], and fearful faces, *p* =.024, *n^2^_p_* = .02, [.002, .065], but not happy, *p* =1.000, *n^2^_p_* = .00, [.00, .00], or sad faces, *p* =.130, *n^2^_p_* = .01, [.00, .04]. Finally, we found a significant interaction between mask use and emotion, *F* (3, 602) = 49.05, *p* < .001, *n^2^_p_* = .20, [.15, .24]. Specifically, the negative impact of mask use on *d*’ was only shown in fearful, happy, and sad faces, *p*s < .001, while angry faces had a similar sensitivity both in masked and unmasked conditions, *p* = .372. There was neither a significant interaction between LSAS and mask use, *F* (1, 602) = 1.21, *p* = .271, *n^2^_p_* = .00, [.00, .01], nor an LSAS x mask use x emotion interaction, *F* (3, 602) = 0.72, *p* = .540, *n^2^_p_* = .00, [.00, .01].

Compared with the above results, our main analysis revealed the relationship between social anxiety and lower false alarm rates for sad faces, providing a more nuanced picture of the relationship between facial emotion sensitivity and social anxiety. This demonstrates the necessity of separately analyzing the accuracy and false alarm rates.

**Figure 1**

*Association Between d’ with LSAS Scores*

##
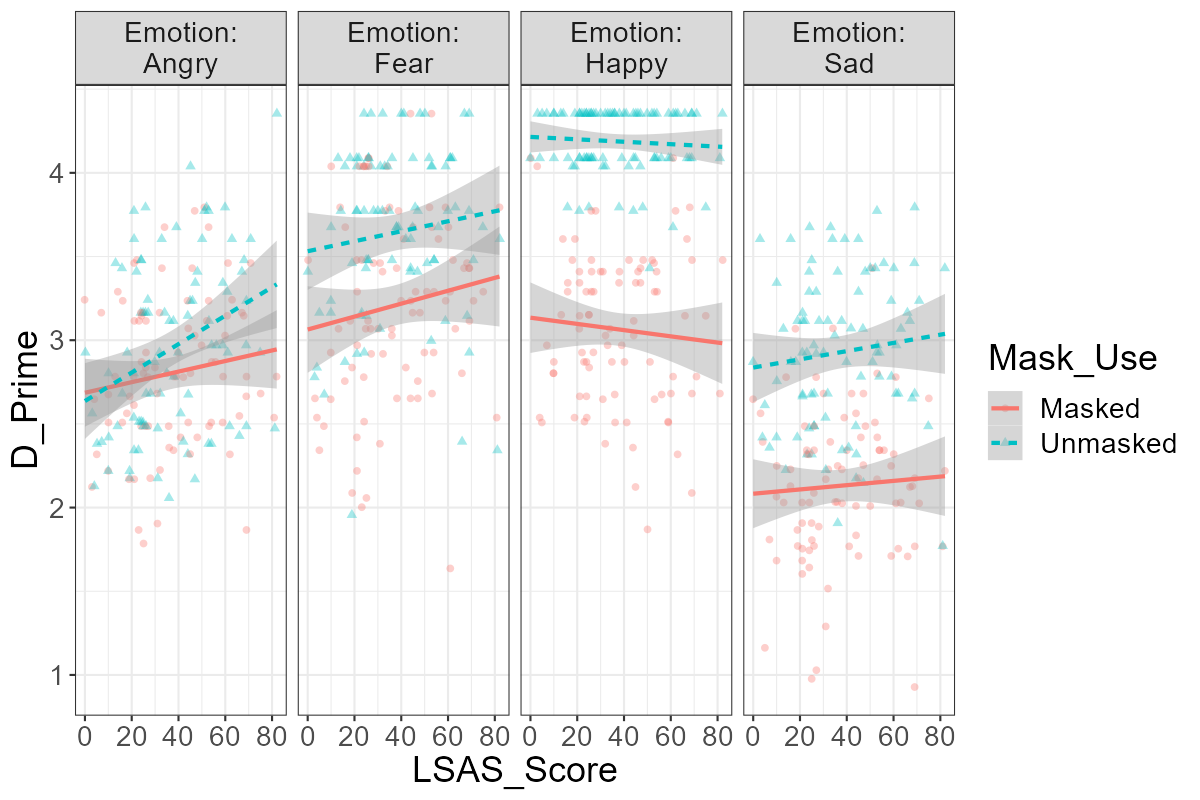


## B. Predefined ROI Approach for Eye Movement Analysis

To understand the difference between the results of eye movement analysis using EMHMM and traditional approaches, following Zheng and Hsiao (2022), here we employed a predefined ROI approach by comparing the frequency of fixations on the predefined eye and nose ROIs (see Figure 2). Similar to the A-B scale, we quantified the fixation percentage difference between the eye and nose regions as: (*E* - *N*) / (|*E*| + |*N*|), where *E* and *N* represent the percentage of fixations to the eye or nose regions respectively. Thus, a more positive E-N fixation percentage difference indicates a higher percentage of fixations on the eye region relative to the nose region. Controlling for the four covariates, the mixed-effect model showed that the relative increase in the percentage of fixations on the eye region due to mask use remained significant, *F* (1, 602) = 388.58, *p* < .001, *n^2^_p_* = .39, [.35, .44]. A significant main effect of emotion was also revealed, *F* (3, 602) = 4.95, *p =* .002, *n^2^_p_* = .02, [.01, .04], and the post-hoc test with Bonferroni correction showed individuals looked at the eyes more for fearful faces compared to angry, *t* (602) = 2.82, *p =* .030, *d* = 0.23, 95% CI [0.07, 0.39], or happy faces, *t* (602) = 3.17, *p* = .009, *d* = 0.26, [0.10, 0.42]. There was no significant main effect of LSAS, *F* (1, 82) = 1.69, *p* = .197, *n^2^_p_* = .02, [.00, .10], or any significant interaction effect: the interaction between LSAS and mask use, *F* (1, 602) = 0.01, *p* = .932, *n^2^_p_* = .00, [.00, .00]; the emotion x LSAS interaction, *F* (3, 602) = 1.09, *p* = .352,  *n^2^_p_* = .01, [.00, .01]; the emotion x mask use interaction, *F* (3, 602) = 1.06, *p* = .366, *n^2^_p_* = .01, [.00, .01]; the interaction between emotion, mask use, and LSAS, *F* (3, 602) = 0.24, *p* = .868, *n^2^_p_* = .00, [.00, .00].

These results generally aligned with the results using EMHMM, showing that mask use increased fixations to the eyes, and social anxiety did not influence participants in adopting fixation strategies across different mask or emotion conditions. The predefined ROI approach revealed a main effect of emotion while the EMHMM approach did not. However, it should be noted that EMHMM summarizes individual differences in both spatial (ROI choices) and temporal (the *transitions* between the ROIs) dimensions of eye movements, and thus provides a more comprehensive, quantitative measure of individual differences in eye movement patterns than the traditional pre-defined ROI approaches.

**Figure 2**

*Predefined Regions of Interest for Eyes and Nose*


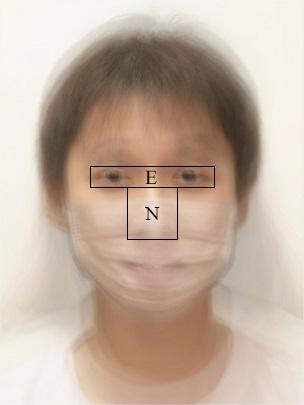


*Notes.* All stimuli were scaled to 304x405 pixels in the *Facial Emotion Recognition Task.* The eye ROI was defined by a rectangular area with the top-left corner located at (90, 166) pixels and the bottom-right corner located at (214, 187) pixels. The nose ROI was defined by a rectangular area with the top-left corner located at (127, 187) pixels and the bottom-right corner located at (177, 239) pixels.

## C. Correlation Matrix Between Recognition Changes and Eye Movement Changes

**Table 1**

*Angry Faces*

|  | |  | | | **ACC Change** | | | **FA Change** | | **RT Change** | | **A-B Scale Change** | |
| --- | --- | --- | --- | --- | --- | --- | --- | --- | --- | --- | --- | --- | --- |
| **ACC Change** |  | | *Spearman's rho* |  | | — |  |  |  |  |  |  |  |
|  |  | | *p-value* |  | | — |  |  |  |  |  |  |  |
| **FA Change** |  | | *Spearman's rho* |  | | .11 |  | — |  |  |  |  |  |
|  |  | | *p-value* |  | | .328 |  | — |  |  |  |  |  |
| **RT Change** |  | | *Spearman's rho* |  | | -.03 |  | -.41 | *** | — |  |  |  |
|  |  | | *p-value* |  | | .754 |  | <.001 |  | — |  |  |  |
| **A-B Scale Change** |  | | *Spearman's rho* |  | | .03 |  | .05 |  | -.15 |  | — |  |
|  |  | | *p-value* |  | | .810 |  | .650 |  | .161 |  | — |  |

*Notes.* Controlling for Gender, DASS Depression Subscore, DASS Stress Subscore, and ToL Preplanning time. * p < .05, ** p< .01, *** p < .001. ACC = accuracy rate; FA = false alarm rate; RT = reaction time of correct trials.

**Table 2**

*Fearful Faces*

|  | |  | | | **ACC Change** | | | **FA Change** | | **RT Change** | | **A-B Scale Change** | |
| --- | --- | --- | --- | --- | --- | --- | --- | --- | --- | --- | --- | --- | --- |
| **ACC Change** |  | | *Spearman's rho* |  | | — |  |  |  |  |  |  |  |
|  |  | | *p-value* |  | | — |  |  |  |  |  |  |  |
| **FA Change** |  | | *Spearman's rho* |  | | .28 | ** | — |  |  |  |  |  |
|  |  | | *p-value* |  | | .009 |  | — |  |  |  |  |  |
| **RT Change** |  | | *Spearman's rho* |  | | .02 |  | -.00 |  | — |  |  |  |
|  |  | | *p-value* |  | | .828 |  | .982 |  | — |  |  |  |
| **A-B Scale Change** |  | | *Spearman's rho* |  | | -.16 |  | .04 |  | .02 |  | — |  |
|  |  | | *p-value* |  | | .149 |  | .718 |  | .892 |  | — |  |

*Notes.* Controlling for Gender, DASS Depression Subscore, DASS Stress Subscore, and ToL Preplanning time. * p < .05, ** p< .01, *** p < .001. ACC = accuracy rate; FA = false alarm rate; RT = reaction time of correct trials.

**Table 3**

*Happy Faces*

|  | |  | | | **ACC Change** | | | **FA Change** | | **RT Change** | | **A-B Scale Change** | |
| --- | --- | --- | --- | --- | --- | --- | --- | --- | --- | --- | --- | --- | --- |
| **ACC Change** |  | | *Spearman's rho* |  | | — |  |  |  |  |  |  |  |
|  |  | | *p-value* |  | | — |  |  |  |  |  |  |  |
| **FA Change** |  | | *Spearman's rho* |  | | .16 |  | — |  |  |  |  |  |
|  |  | | *p-value* |  | | .137 |  | — |  |  |  |  |  |
| **RT Change** |  | | *Spearman's rho* |  | | .10 |  | -.01 |  | — |  |  |  |
|  |  | | *p-value* |  | | .368 |  | .948 |  | — |  |  |  |
| **A-B Scale Change** |  | | *Spearman's rho* |  | | .02 |  | -.05 |  | .17 |  | — |  |
|  |  | | *p-value* |  | | .863 |  | .638 |  | .115 |  | — |  |

*Notes.* Controlling for Gender, DASS Depression Subscore, DASS Stress Subscore, and ToL Preplanning time. * p < .05, ** p< .01, *** p < .001. ACC = accuracy rate; FA = false alarm rate; RT = reaction time of correct trials.

**Table 4**

*Sad Faces*

|  | |  | | | **ACC Change** | | | **FA Change** | | **RT Change** | | **A-B Scale Change** | |
| --- | --- | --- | --- | --- | --- | --- | --- | --- | --- | --- | --- | --- | --- |
| **ACC Change** |  | | *Spearman's rho* |  | | — |  |  |  |  |  |  |  |
|  |  | | *p-value* |  | | — |  |  |  |  |  |  |  |
| **FA Change** |  | | *Spearman's rho* |  | | .18 |  | — |  |  |  |  |  |
|  |  | | *p-value* |  | | .096 |  | — |  |  |  |  |  |
| **RT Change** |  | | *Spearman's rho* |  | | -.21 |  | -.10 |  | — |  |  |  |
|  |  | | *p-value* |  | | .059 |  | .372 |  | — |  |  |  |
| **A-B Scale Change** |  | | *Spearman's rho* |  | | -.14 |  | -.10 |  | .10 |  | — |  |
|  |  | | *p-value* |  | | .191 |  | .364 |  | .374 |  | — |  |

*Notes.* Controlling for Gender, DASS Depression Subscore, DASS Stress Subscore, and ToL Preplanning time. * p < .05, ** p< .01, *** p < .001. ACC = accuracy rate; FA = false alarm rate; RT = reaction time of correct trials.

# References

Macmillan, N. A., & Kaplan, H. L. (1985). Detection theory analysis of group data: Estimating sensitivity from average hit and false-alarm rates. *Psychological Bulletin*, *98*(1), 185–199. <https://doi.org/10.1037/0033-2909.98.1.185>

Zheng, Y., & Hsiao, J. H. (2023). Differential audiovisual information processing in emotion recognition: An eye-tracking study. *Emotion, 23*(4), 1028–1039. <https://doi.org/10.1037/emo0001144>

1. *d*′ = *Z*(*Accuracy Rate*) − *Z*(*False Alarm Rate*) in the present study. In the calculation of *d*’, the extreme values of accuracy and false alarm rates were adjusted by replacing rates of 0 with .5/*n* and 1 with (*n -* .5)/*n*, in which *n* represents the number of signal or noise trials (Macmillan & Kaplan, 1985). [↑](#footnote-ref-2)
